# Comparison of the effectiveness of chemotherapy combined with immunotherapy and chemotherapy alone in advanced biliary tract cancer and construction of the nomogram for survival prediction based on the inflammatory index and controlling nutritional status score

**DOI:** 10.1007/s00262-023-03513-4

**Published:** 2023-09-05

**Authors:** Zhengfeng Zhang, Dazhen Wang, Jianji Zhang, Yuli Ruan, Lu Zhao, Liu Yang, Ze Liu, Lei Yang, Changjie Lou

**Affiliations:** 1 Department of Gastroenterology, Harbin Medical University Cancer Hospital, 150 Haping Road, Nangang District, 150081 Heilongjiang Province China; 2 Mianyang Central Hospital, Mianyang, 621000 China

**Keywords:** Nomogram, Advanced biliary tract cancer, Immune checkpoint inhibitors, Inflammatory index, Controlling nutritional status score

## Abstract

**Objective:**

To analyze the effectiveness of combining immune checkpoint inhibitors (ICIs) with first-line therapy in patients with advanced biliary tract cancer (BTC) and explore the biomarkers affecting the prognosis of immunotherapy, to construct a nomogram for the prediction of survival.

**Methods:**

A retrospective study was conducted to include a total of 209 patients with advanced BTC treated in the first line from 2018 to 2022, divided into a combination therapy group (*n* = 129) and a chemotherapy-only group (*n* = 80) according to whether ICIs were applied in combination. Univariate and multifactorial COX regression analyses were performed on variables that may affect prognosis to identify independent influences on patient prognosis, and this was used to create nomograms, which were then prospectively validated and calibrated.

**Results:**

The median progression-free survival (mPFS) and median overall survival (mOS) of patients in the combination therapy group were higher than those in the chemotherapy alone group [hazard ratio (HR) = 1.152, 95% confidence interval (CI): 0.7848–1.692, *p* = 0.0004, and HR = 1.067, 95% CI: 0.7474–1.524, *p* = 0.0016]. The objective response rate (ORR) of patients in the combination therapy and chemotherapy alone groups was 39.5% (51/129) vs. 27.5% (22/80), and the disease control rate (DCR) between the two groups was 89.9% (116/129) vs. 83.8% (67/80). Univariate analysis revealed the gender, presence of long-term tobacco and alcohol, degree of histological differentiation, serum albumin level, presence of liver metastases, presence of multi-visceral metastases, response, neutrophil-to-lymphocyte ratio (NLR), platelet-to-lymphocyte ratio (PLR), monocyte-to-lymphocyte ratio (MLR), glycoprotein antigen 19-9 (CA19-9), systemic inflammatory index (SII), and controlling nutritional status (CONUT) scores were statistically significant with patient prognosis (all *P* values < 0.05). Multi-factor COX regression analysis was continued for the above variables, and the results showed that NLR, MLR, PLR, SII, and CONUT scores were independent influences on patients’ OS (all *p* values < 0.05). A nomogram (C-index 0.77, 95% CI: 0.71–0.84) was created based on these independent influences and later validated using a validation cohort (C-index 0.75, 95% CI: 0.68–0.81). The time-dependent receiver operator characteristic curve (ROC) showed that the area under curve (AUC) of the training cohort patients at 12, 18, and 24 months was 0.72 (95% CI: 0.63–0.81), 0.75 (95% CI: 0.67–0.85), and 0.77 (95% CI: 0.66–0.87) and the AUC of the validation cohort was 0.69 (95% CI: 0.58–0.79), 0.74 (95% CI: 0.65–0.87), and 0.71 (95% CI: 0.64–0.89), respectively. Finally, calibration was performed using calibration curves, and the results showed that nomograms based on inflammatory metrics and CONUT scores could be used to assess survival (12, 18, and 24 months) in patients with advanced BTC treated with ICIs in the first line.

**Conclusion:**

Patients with advanced BTC benefit more from first-line treatment with standard chemotherapy in combination with ICIs than with chemotherapy alone. In addition, nomograms based on inflammatory metrics and CONUT scores can be used to predict survival at 12, 18, and 24 months in patients with advanced BTC treated with ICIs.

**Supplementary Information:**

The online version contains supplementary material available at 10.1007/s00262-023-03513-4.

## Introduction

Biliary tract cancer (BTC) is a malignant tumor originating from biliary tract epithelial cells, accounting for about 3% of GI malignancies, which can be divided into cholangiocarcinoma (CCA) and gallbladder carcinoma (GCA) according to the site of development, and the CCA can be further divided into intrahepatic cholangiocarcinoma (ICCA), hilar cholangiocarcinoma (HCC), and distal cholangiocarcinoma (DCCA) [1, 2]. BTC has an aggressive biological behavior, and most patients are diagnosed at an advanced stage; therefore, the prognosis is often poor, with a 5-year survival rate of less than 10% and a median survival of less than 1 year. [3–5]. The current treatment of advanced BTC is based on chemotherapy with Gemcitabine as the cornerstone, supplemented by targeted therapy, hepatic artery embolization chemotherapy, radiotherapy, and other therapeutic measures. Although immunotherapy based on ICIs has achieved better results in several solid tumor species, the evidence for its application in BTC is not yet sufficient [6, 7]. In the KEYNOTE-028 and KEYNOTE-224 clinical trials, single-agent pembrolizumab showed some benefits; in addition, in the KEYNOTE-158 clinical trial, patients with advanced BTC with positive PD-L1 expression achieved an ORR of 40.9% with pembrolizumab, and even 17% of patients achieved PR, but most studies mainly recommended single-agent pembrolizumab for second-line or even later treatment in BTC patients who failed first-line therapy [4, 8, 9]. Regarding the efficacy and safety of immune combination therapy in the first-line treatment of patients with advanced BTC, several phase III clinical trials have been conducted, of which only the phase III clinical trial of TOPAZ-1 met the study endpoint, and the results of this study showed that durvalumab in combination with standard chemotherapy was beneficial in terms of both overall survival (OS) and progression-free survival (PFS) in patients with advanced BTC and showed good safety and tolerability [10]. Other phases III clinical studies have not yet met their endpoints, so the evidence for the use of ICIs for the first-line treatment of patients with advanced BTC is still insufficient, so we conducted this retrospective study.

Recently, it has been found that changes in inflammation-related indicators are crucial for tumorigenesis and progression, such as NLR, PLR, MLR, SII, and other indicators be of significant value in the prognosis of esophageal, cervical, pancreatic, and hepatocellular carcinomas [11–14]. The CONUT score is also a recently developed biomarker of inflammation and nutritional status based on serum albumin, total cholesterol, and peripheral blood lymphocyte count, has been used in the prognostic assessment of gastric, esophageal, colorectal, and lung cancers, and has shown some feasibility [15–18]. However, less is known about the application of CONUT scores and inflammatory indicators in predicting and assessing long-term survival in patients with advanced BTC, especially in advanced patients receiving first-line immunotherapy; therefore, we conducted this retrospective study to construct nomograms to predict patient survival while analyzing the efficacy assessment of immune combination chemotherapy for first-line treatment of advanced BTC.

## Materials and methods

### Inclusion and exclusion criteria

A total of 309 patients with advanced BTC treated at the Harbin Medical University Cancer Hospital from 2018 to 2022 were included in the study. A total of 209 patients achieved PFS or OS (129 in combination with immunotherapy and 80 in the chemotherapy alone), and a total of 100 patients received immunotherapy in combination but did not achieve PFS or OS. The main inclusion criteria were: (1) pathological diagnosis of BTC (including cholangiocarcinoma and gallbladder cancer), (2) presence of local progression or distant metastasis, (3) presence of measurable target lesions, (4) no combination of other primary tumors, and (5) complete clinical record data. The exclusion criteria: (1) non-advanced BTC patients, (2) no measurable target lesions, (3) combination of other active tumors, and (4) incomplete medical records.

### General information

The following variables were collected and analyzed: gender, age, height, weight, history of smoking and alcohol consumption, site of primary focus, histological classification, Eastern Cooperative Oncology Group (ECOG) score, whether ICIs were combined and the different types of ICIs, time of first definitive diagnosis, radical surgery, postoperative adjuvant chemotherapy, whether combined with radiotherapy, whether radiofrequency ablation or hepatic artery infusion chemotherapy was performed, distant metastases, hematological findings (white blood cell count, neutrophil count, platelet count, hemoglobin count, lymphocyte count, total cholesterol, serum albumin, tumor markers, etc.), imaging findings, the response of tumor, time of disease progression and reasons for progression, survival of patients at the last follow-up, time of death and the cause of death, etc. Preoperative CONUT scores were calculated from the serum albumin concentration, total lymphocyte count, and cholesterol concentration data and are shown in Table 1.

**Table 1:** Assessment of nutritional status according to CONUT score

Variables	Range	Score
Serum albumin (g/dL)	≥ 3.5	0
	3–3.49	2
	2.5–2.99	4
	< 2.5	6
Total cholesterol (mg/dL)	≥ 180	0
	140–179	1
	100–139	2
	< 100	3
Lymphocyte count (10^9/L**)**	≥ 1.6	0
	1.2–1.59	1
	0.8–1.19	2
	< 0.8	3

*CONUT* controlling nutritional status. The CONUT score is the sum of serum albumin, total cholesterol, and peripheral blood lymphocyte count. We chose the optimal cutoff value of the CONUT score as 2 and divided patients into the low CONUT score group (CONUT < 2) and the high CONUT score group (CONUT ≥ 2)

Follow-up was performed by reviewing inpatient case information, follow-up visits, and telephone contact, with the last follow-up up to September 2022. The time from the start of the first standard treatment until the patient progresses or dies is called PFS, and the time from the start of standard treatment until death (from any cause) is OS.

### Efficacy evaluation standard

Both the combination therapy and chemotherapy alone groups were performed according to Response Evaluation Criteria In Solid Tumors (RECIST1.1); they can be classified as complete response (CR), partial response (PR), stable disease (SD), and progressive disease (PD). DCR is the proportion of cases other than PD after drug administration, and ORR is the percentage of patients with the best efficacy rating of CR and PR in the total number of effective cases during treatment.

### Statistical methods

We used SPSS 26.0 software, R software version 4.2.1, and GraphPad prism 9.0 software for statistical analysis and plotting of the data. Continuous measures obeying normal distribution were expressed as mean ± standard (mean ± SD) deviation; one-way ANOVA was used for comparison between groups of measures obeying normal distribution with uniform variance, and LSD was used for two-way comparison; the Kruskal–Wallis test was used for those not obeying normal distribution with an uneven variance; Chi-square test was used for comparison between groups of count data, and utilization rate or composition ratio was expressed. The Pearson linear correlation was used for bivariate normal distribution, and the Spearman rank correlation was used for non-normal distribution; the Cox regression analysis was used for multi-factor analysis and made *p* < 0.05 as the difference was statistically significant. The 209 patients who had achieved OS were divided into a combination treatment group (*n* = 129) and a chemotherapy alone group (*n* = 90) according to the treatment regimen for effectiveness analysis, and the Kaplan–Meier method was used to plot survival curves and to compare survival by log-rank test. Univariate and multifactorial regression analyses were performed on 129 patients in the combined treatment group, and then, the training cohort was included for COX regression modeling based on the results of the multifactorial analyses, and the nomogram was plotted and the C-index was calculated using R software. The patients in the validation cohort were not from training cohort, further 100 immunotherapy patients were included as the validation cohort, and the constructed functional model was validated using ROC curves and AUC, calibration curves. All tests were bivariate, and the results with a *P* value < 0.05 were considered statistically significant.

## Results

### Basic characteristics of the combination therapy group and chemotherapy alone group

A total of 209 patients with advanced BTC treated first-line data were included in the study, including 129 patients in the combination therapy group, 81 patients (62.8%) with bile duct cancer and 25 patients (37.2%) with gallbladder cancer, and 80 patients in the chemotherapy alone group, 65 patients (81.3%) with bile duct cancer and 15 patients (18.7%) with gallbladder cancer, all with ECOG scores of 0–1. There was no statistical difference in age, gender, height, weight, primary tumor location, previous history of smoking and alcohol, total bilirubin, ALT, and AST between the patients in the combination treatment group and the chemotherapy alone group (*p* > 0.05). There were more men than women in both the combination therapy group and the chemotherapy alone group. Statistical differences existed between the two groups in terms of NLR, PLR, whether ICIs were used in combination, degree of tumor differentiation and the presence of multisite metastasis (*p* < 0.05) (Table 2).

**Table 2:** Baseline characteristics between ICIs combined with chemotherapy group and chemotherapy alone group

Characteristics	ICIs + chemotherapy n = 129	Chemotherapy n = 80	*P* value
Combine with ICIs	Yes	No	< 0.001
Gender			0.082
Male	81 (62.8)	42 (52.5)	
Female	48 (37.2)	38 (47.5)	
Age (years)	60.12 ± 8.99	60 ± 9.33	0.061
< 60	59 (45.7)	38(47.5)	
≥ 60	70 (54.3)	42 (52.5)	
Smoking or drinking			0.124
Yes	68 (52.7)	34 (42.5)	
No	61 (47.3)	46(57.5)	
Tumor location			0.077
ICC	61 (47.3)	34(42.4)	
HCCA	25 (19.8)	16(20.0)	
DCCA	18 (13.1)	15(18.8)	
GCA	25 (19.8)	15 (18.8)	
Tumor differentiation			0.042
Well	7 (5.4)	4(5.0)	
Moderately	37 (28.7)	21 (26.3)	
Poorly	36 (27.9)	23 (28.8)	
Unknown	49 (38.0)	32 (39.9)	
Serum albumin			0.100
< 36 g/L	77 (59.7)	44(55.0)	
≥ 36 g/L	52 (40.3)	36(45.0)	
Liver metastasis			0.055
Yes	81 (62.8)	42 (52.5)	
No	48 (37.2)	38 (47.5)	
Multi-site metastasis			0.031
Yes	77 (59.7)	43 (53.8)	
No	52 (40.3)	37(46.2)	
Response			0.042
CR	10 (7.8)	1 (1.3)	
PR	41 (31.8)	21(26.3)	
SD	65 (50.4)	45 (56.3)	
PD	13 (10.0)	13 (16.1)	
NLR			0.021
< 3.0	57 (44.2)	37 (46.3)	
≥ 3.0	72 (55.8)	43 (53.7)	
PLR			0.032
< 160	58 (45.0)	35(43.8)	
≥ 160	71 (55.0)	45(56.2)	
Total bilirubin			0.103
< 21umol/L	34 (26.4)	29 (36.6)	
≥ 21umol/L	95 (73.6)	51(63.4)	
ALT			0.241
< 50U/mL	61 (47.3)	41 (51.3)	
≥ 50U/mL	68 (52.7)	39(48.7)	
CA19-9			0.002
< 37U/mL	42 (32.6)	30(37.5)	
≥ 37U/mL	87 (67.4)	50(62.5)	
AST			0.334
< 40U/mL	48 (37.2)	32(40.0)	
≥ 40U/mL	81 (62.8)	48(60.0)	
MLR			0.007
< 2.3	60 (46.5)	33(41.3)	
≥ 2.3	69 (53.5)	47(58.7)	
SII			0.015
< 830.06	58 (45.0)	29(36.3)	
≥ 830.06	71 (55.0)	51 (63.7)	
CONUT score			0.144
< 2	59 (45.7)	34 (42.5)	
≥ 2	70 (54.3)	46(57.5)	

*ICCA* intrahepatic cholangiocarcinoma; *HCCA* hilar cholangiocarcinoma; *DCCA* distal cholangiocarcinoma; *GCA* gallbladder carcinoma; *NLR* neutrophil-to-lymphocyte ratio; *PLR* platelet-to-lymphocyte ratio; *MLR* monocyte-to-lymphocyte ratio; *SII* systemic inflammatory index (SII was calculated by multiplying the platelet count by the neutrophil count divided by the lymphocyte count); *ALT* alanine transaminase; *AST* aspartate transaminase; *CA19-9* glycoprotein antigen 19-9; *CONUT score* controlling nutritional status score

### Analysis of treatment effectiveness

**Fig. 1:**
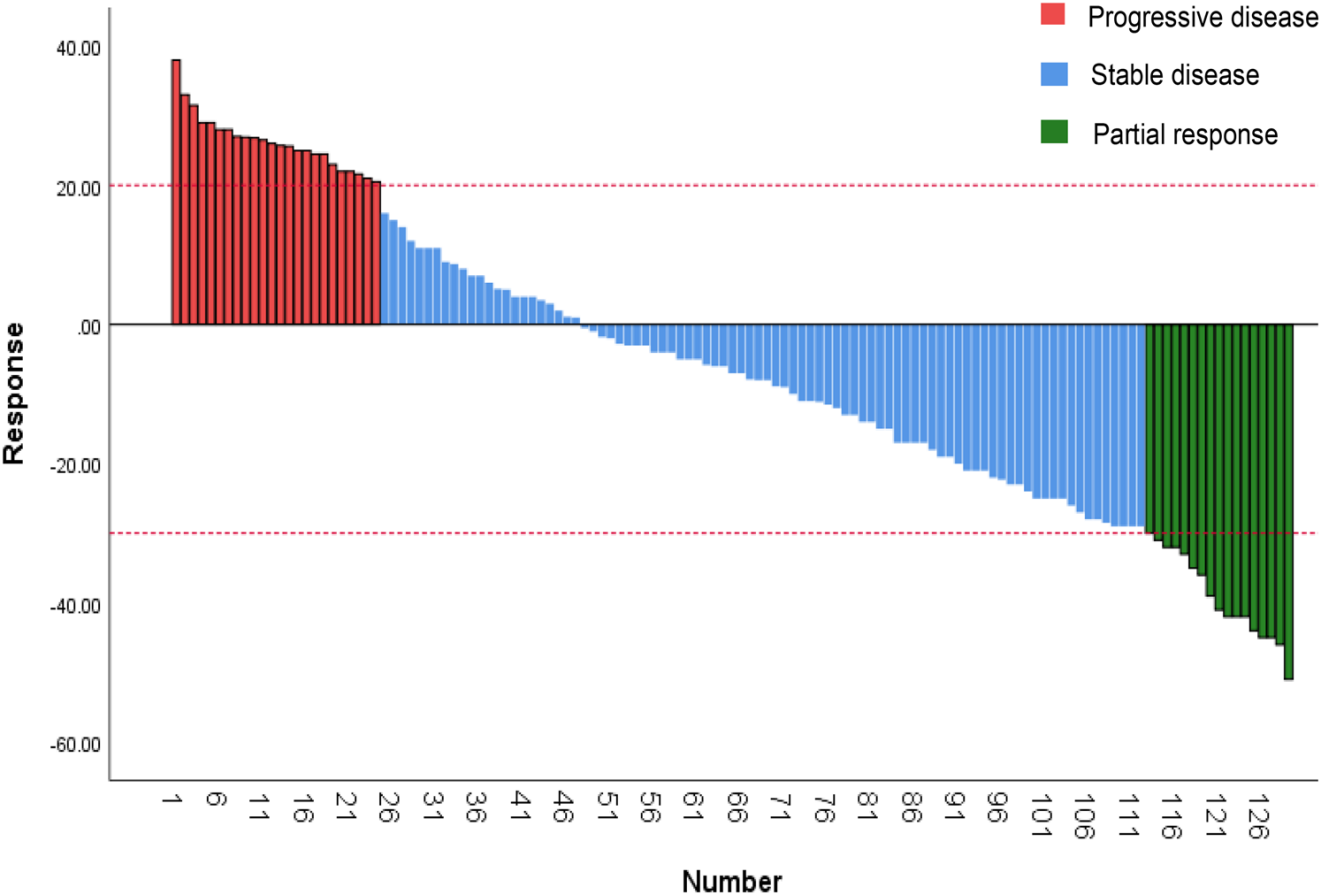
Waterfall plot of the combination immunotherapy treatment group

A total of 10 patients (7.8%) in the combination treatment group had an efficacy evaluation of CR, 41 patients (31.8%) had an evaluation of PR, 65 patients (50.4%) had an evaluation of SD, and 13 patients (10.1%) had an evaluation of PD (Fig. 1). The ORR of patients in the combination treatment group was 39.5% (51/129) and the DCR was 89.9% (116/129). One patient (1.2%) in the chemotherapy alone group had a CR, 21 patients (26.3%) had an efficacy evaluation of PR, 45 patients (56.3%) had an efficacy evaluation of SD, and 13 (16.3%) had an efficacy evaluation of PD. The ORR of patients in the chemotherapy alone group was 27.5% (22/80) and the DCR was 83.8% (67/80).

**Fig. 2:**
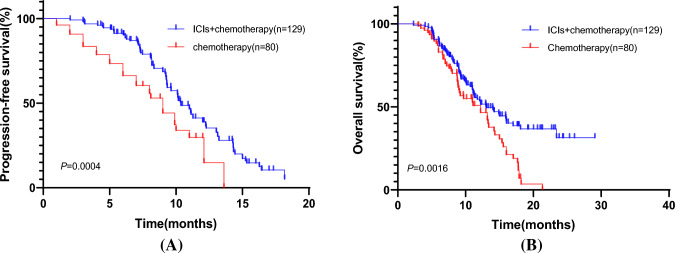
Kaplan–Meier survival curve of two groups: **A** progression-free survival time of two groups, **B** overall survival of two groups

The overall ORR and DCR of patients in the combination treatment group were higher than those in the chemotherapy alone group, 39.5% (51/129) vs. 27.5% (22/80) and 89.9% (116/129) vs. 83.8% (67/80), respectively, with statistically significant differences (*p* < 0.05). Patients in the chemotherapy combined with ICIs group had higher progression-free survival (PFS) and overall survival (OS) than those in the chemotherapy alone group [hazard ratio (HR) = 1.152, 95% confidence interval (CI): 0.7848–1.692, *P* = 0.0004 and HR = 1.067, 95% CI: 0.7474–1.524, *p* = 0.0016] (Fig. 2).

### Basic characteristics of training cohort and validation cohort

We included 129 patients in the combination therapy group in the training cohort and randomly selected 100 patients currently receiving immune combination therapy as the validation cohort. A total of 229 patients included in the study were not statistically different in terms of gender, age, smoking and alcohol history, primary tumor site, degree of histological differentiation, NLR, PLR, MLR, SII, ECOG score, CA19-9, and CONUT score (*p* > 0.05). In both the training cohort and the validation cohort, there were more men than women, more patients with a history of smoking or drinking than without a history of smoking or drinking, significantly more bile duct cancer than gallbladder cancer, and the majority of patients in both groups were over 60 years of age (Table 3).

**Table 3:** Training cohort and validation cohort baseline characteristics

Characteristics	Entire cohort N = 229	Training cohort N1 = 129	Validation cohort N2 = 100	*P* value
Gender				0.062
Male	145 (63.3)	81 (62.8)	64 (64.0)	
Female	84 (36.7)	48 (37.2)	36 (36.0)	
Age (years)	60.42 ± 7.17	60.12 ± 8.99	59.38 ± 10.19	0.728
< 60	103 (45.0)	59 (45.7)	44 (44.2)	
≥ 60	126 (55.0)	70 (54.3)	56 (55.8)	
Smoking or drinking				0.324
Yes	120 (52.4)	68 (52.7)	52 (52.3)	
No	109 (47.6)	61 (47.3)	48 (47.7)	
Tumor location				0.134
ICC	109 (47.6)	61 (47.3)	48 (47.6)	
HCCA	45 (19.7)	25 (19.8)	20 (19.8)	
DCCA	32 (14.0)	18 (13.1)	14 (14.0)	
GCA	43 (18.7)	25 (19.8)	18 (18.6)	
Tumor differentiation				0.224
Well	13 (5.7)	7 (5.4)	6 (5.8)	
Moderately	67 (29.3)	37 (28.7)	30 (30.2)	
Poorly	65 (28.4)	36 (27.9)	29 (29.1)	
Unknown	84 (36.6)	49 (38.0)	35 (34.9)	
Serum albumin				0.336
< 36 g/L	137 (59.8)	77 (59.7)	60 (60.5)	
≥ 36 g/L	92 (40.2)	52 (40.3)	40 (39.5)	
Liver metastasis				0.205
Yes	139 (60.7)	81 (62.8)	58 (58.1)	
No	90 (39.3)	48 (37.2)	42 (41.9)	
Multi-site metastasis				0.084
Yes	130 (56.8)	77 (59.7)	53 (53.5)	
No	99 (43.2)	52 (40.3)	47 (46.5)	
Types of immune drugs				0.718
Camrelizumab	49 (21.3)	28 (21.7)	21 (20.9)	
Pembrolizumab	51 (22.3)	30 (23.3)	21 (20.9)	
Toripalimab	62 (20.1)	36 (27.9)	26 (25.6)	
Others	67 (29.3)	35 (27.1)	32 (32.6)	
Response				0.077
CR	18 (7.9)	10 (7.8)	8 (8.1)	
PR	75 (32.8)	41 (31.8)	34 (33.7)	
SD	111 (48.5)	65 (50.4)	46 (46.5)	
PD	25 (10.8)	13 (10.0)	12 (11.7)	
NLR				0.321
< 3.0	100 (43.7)	57 (44.2)	43 (43.0)	
≥ 3.0	129 (56.3)	72 (55.8)	57 (57.0)	
PLR				0.068
< 160	99 (43.2)	58 (45.0)	41 (40.7)	
≥ 160	130 (56.8)	71 (55.0)	59 (59.3)	
Total bilirubin				0.144
< 21umol/L	60 (26.2)	34 (26.4)	26 (25.6)	
≥ 21umol/L	169 (73.8)	95 (73.6)	74 (74.4)	
ALT				0.101
< 50U/mL	109 (47.6)	61 (47.3)	48 (47.6)	
≥ 50U/mL	120 (52.4)	68 (52.7)	52 (52.4)	
CA19-9				0.331
< 37U/mL	84 (36.7)	42 (32.6)	42 (42.6)	
≥ 37U/mL	145 (63.3)	87 (67.4)	58 (58.1)	
AST				0.057
< 40U/mL	83 (36.2)	48 (37.2)	35 (34.9)	
≥ 40U/mL	146 (63.8)	81 (62.8)	65 (65.1)	
MLR				0.237
< 2.3	108 (47.2)	60 (46.5)	48 (47.6)	
≥ 2.3	121 (52.8)	69 (53.5)	52 (52.4)	
SII				0.054
< 830.06	103 (45.0)	58 (45.0)	45 (45.3)	
≥ 830.06	126 (55.0)	71 (55.0)	55 (54.7)	
CONUT score				0.104
< 2	101 (44.1)	59 (45.7)	42 (41.9)	
≥ 2	128 (55.9)	70 (54.3)	58 (58.1)	

*ICCA* intrahepatic cholangiocarcinoma; *HCCA* hilar cholangiocarcinoma; *DCCA* distal cholangiocarcinoma; *GCA* gallbladder carcinoma; *NLR* neutrophil-to-lymphocyte ratio; *PLR* platelet-to-lymphocyte ratio; *MLR* monocyte-to-lymphocyte ratio; *SII* systemic inflammatory index (SII was calculated by multiplying the platelet count by the neutrophil count divided by the lymphocyte count); *ALT* alanine transaminase; *AST* aspartate transaminase; *CA19-9* glycoprotein antigen 19-9; *CONUT score* controlling nutritional status score; other types of immune drugs included sintilimab and dovalizumab.

**Fig. 3:**
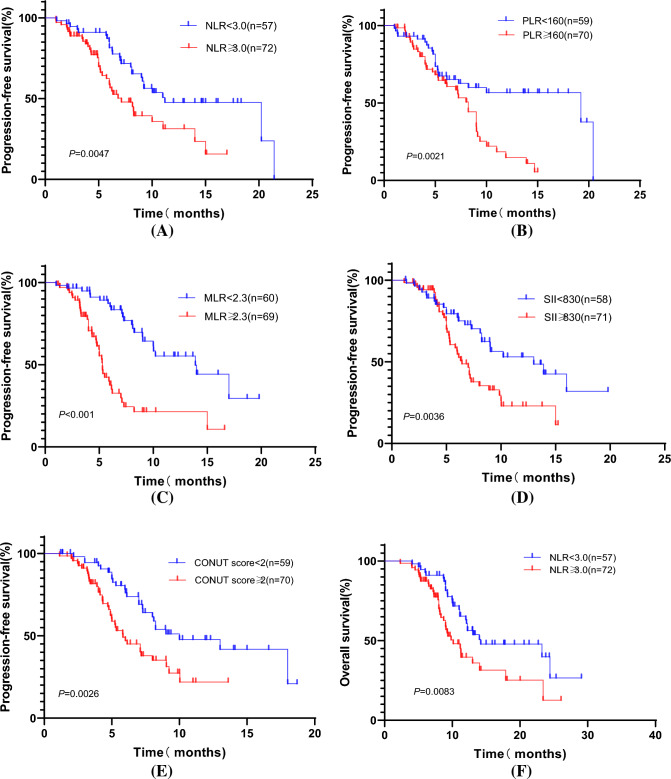

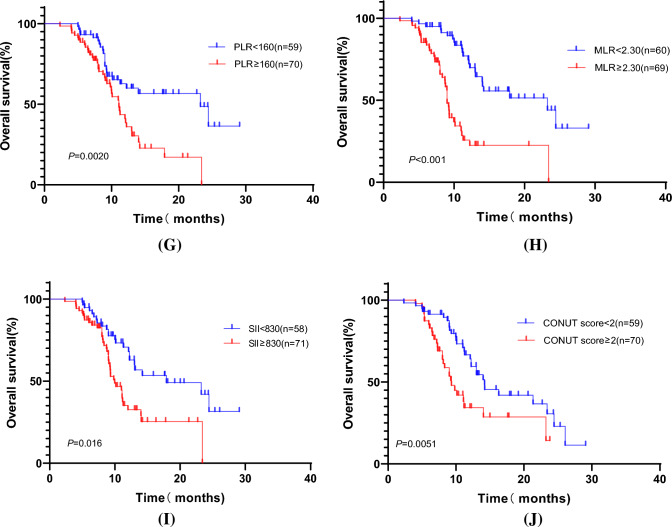
Progression-free survival (PFS) and overall survival (OS) curves in advanced BTC patients of training cohort. **A** PFS according to the NLR; **B** PFS according to the PLR; **C** PFS according to the MLR; **D** PFS according to the SII value; **E** PFS according to the CONUT score; **F** OS according to the NLR; **G** OS according to the PLR; **H** OS according to the MLR; **I** OS according to the SII value; and **J** OS according to the CONUT score

We performed a univariate regression analysis of the variables in the training cohort and found that patients' gender, previous history of smoking and alcohol, degree of tumor differentiation, presence of liver metastases, presence of multisite metastases, best response, NLR, PLR, MLR, CONUT score, SII, serum albumin level, and CA19-9 value were all correlated with patient prognosis (all *p* < 0.05). We proceeded to include the above factors in a multifactorial COX regression analysis, which showed that lower inflammatory indexes (NLR, PLR, MLR, SII) and CONUT scores (Fig. 3) were associated with better OS in patients (*p* < 0.05) and were independent influencers of OS (Supplementary Table).

### Construction and validation of nomogram

**Fig. 4:**
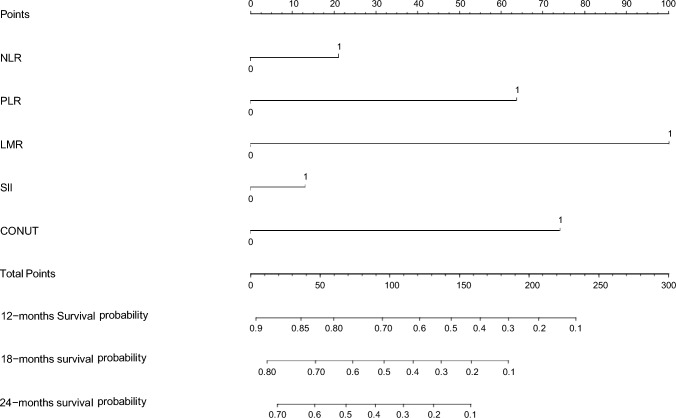
Nomogram to predict the probability of 12-, 18-, and 24-month overall survival (OS) including the NLR (0: NLR < 3.0, 1:NLR ≥ 3.0), PLR (0:PLR < 160, 1:PLR ≥ 160), MLR(0:MLR < 2.3, 1:MLR ≥ 2.3), SII(0:SII < 830, 1:SII ≥ 830), and the CONUT score (0: CONUT score < 2, 1:CONUT score ≥ 2). The nomogram can be used to obtain the probability of 12-, 18, and 24-month OS by adding up the points identified on the point scale for each variable. *Notes*: CA19-9: carbohydrate antigen 19–9; COUNT: controlling nutritional status, NLR: neutrophil-to-lymphocyte ratio; PLR: platelet-to-lymphocyte ratio; MLR: monocyte-to-lymphocyte ratio; SII: systemic inflammatory index.

A total of 229 patients included in the study were divided into two cohorts, 56.3% (*n* = 129) in the training cohort and 43.7% (*n* = 100) in the validation cohort, with no statistical differences between the two groups (all *p* > 0.05). Based on five independent prognostic factors affecting OS, a nomogram was generated in the training cohort to predict OS at 12, 18, and 24 months in patients with advanced BTC receiving first-line combination therapy with ICIs (Fig. 4).

**Fig. 5:**
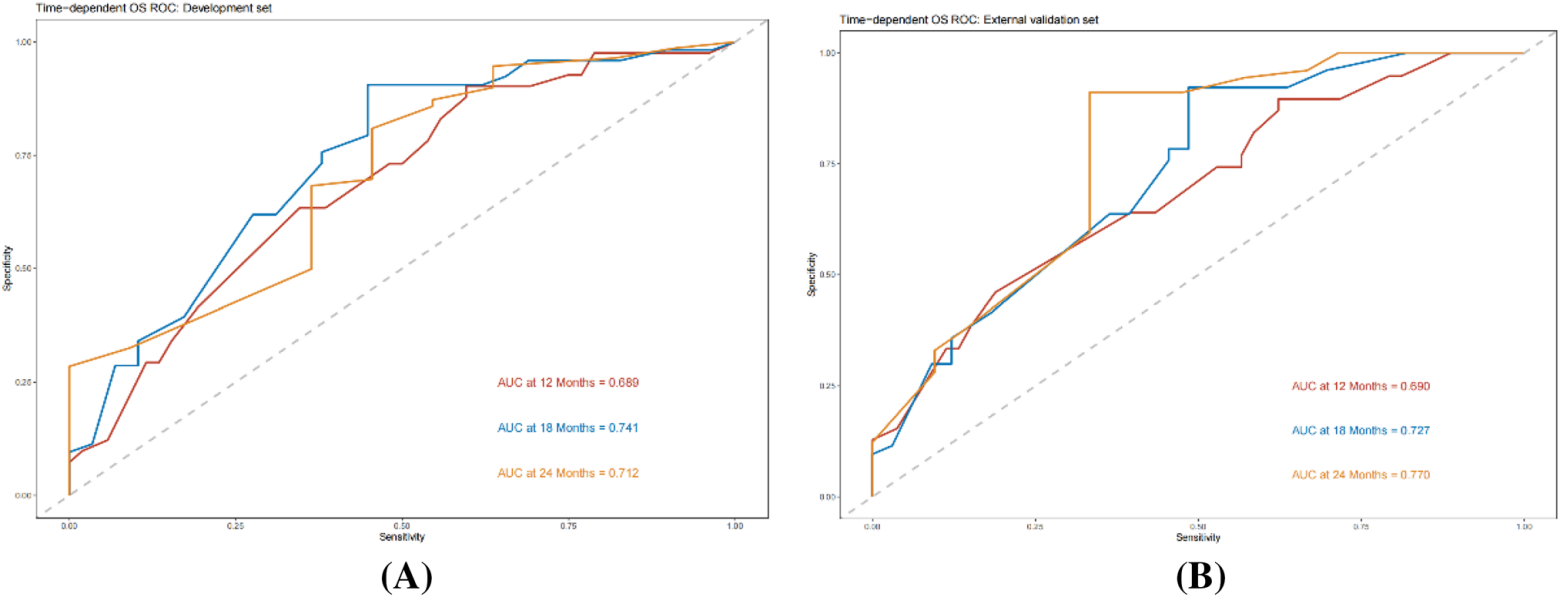
Time ROC curve analyses to compare the predictive performance. **A** ROC curve analyses of 12-, 18-, and 24-month OS in the training cohort; **B** ROC curve analyses of 12-, 18-, and 24-month OS in the training cohort validation cohort

**Fig. 6:**
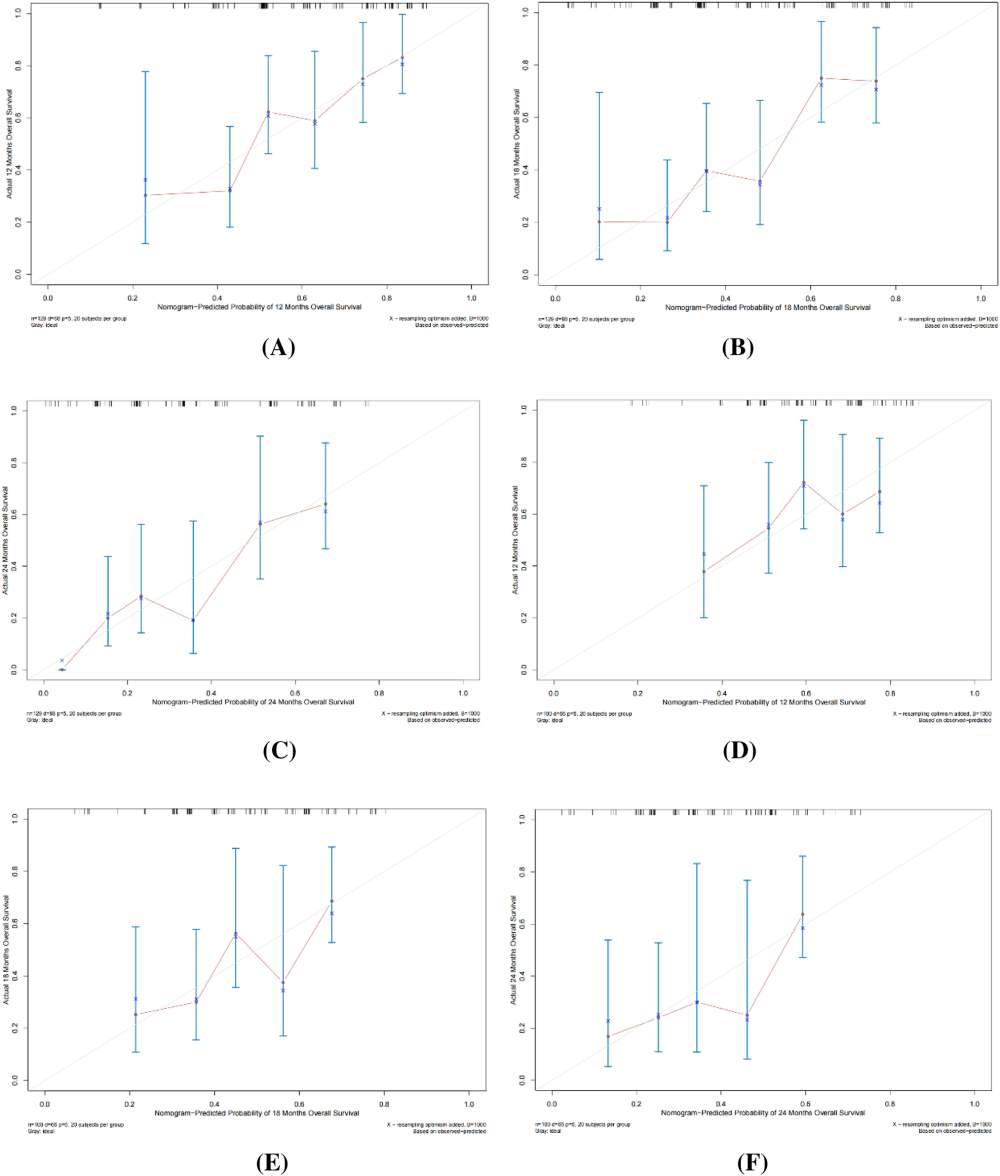
Calibration curve of the nomogram both in the training and validation cohorts. **A** 2-month OS in the training cohort, **B** 18-month OS in the training cohort, **C** 24-month OS in the training cohort; **D** 12-month OS in the validation cohort, **E** 18-month OS in the validation cohort, **F** 24-month OS in the validation cohort

In the training cohort, the C-index of the nomogram was 0.771 (*p* = 0.0012, HR = 0.732, 95% CI: 0.693–0.797), and the C-index of the validation cohort was 0.715 (*p* = 0.0038, HR = 0.701, 95% CI: 0.663–0.747), which was in good agreement with the actual OS. In addition, the AUCs for the training cohort were 0.689 (95% CI:0.627–0.809), 0.741 (95% CI:0.663–0.846), and 0.712 (95% CI:0.661–0.781) at 12, 18, and 24 months, respectively (Fig. 5A). The AUCs at 12, 18, and 24 months for the validation cohort were 0.690 (95% CI:0.589–0.791), 0.727 (95% CI:0.649–0.867), and 0.770 (95% CI:0.635–0.843), respectively (Fig. 5B) (all *p* < 0.05). Calibration plots of OS probabilities at 12, 18, and 24 months for patients with advanced BTC showed a high degree of agreement between the actual survival predictions of the training and validation cohorts (Fig. 6).

## Discussion

We found in this retrospective study that patients with advanced BTC benefited more from the use of ICIs in combination with standard chemotherapy compared with chemotherapy alone at first-line treatment. In our study, the mPFS and mOS of patients in the combination therapy group were 6.37 months and 9.43 months, respectively, which were higher than those in the chemotherapy-only group; in addition, the ORR of 39.5% and DCR of 89.9% were also significantly higher in the combination therapy group compared with 27.5% and 83.8% of patients in the chemotherapy-only group. The results of this study showed full consistency with the TOPAZ-1 phase III trial and even better results than those obtained with TOPAZ-1, and in the subgroup analysis of TOPAZ-1, it was also indicated that Asian patients benefited more from treatment with durvalumab in combination with chemotherapy [19, 20]. In addition, we also found that in this retrospective study we conducted, the ORR of patients in the combination therapy group was also significantly improved compared to the KEYNOTE-028 and KEYNOTE-224 clinical trials of single-agent immunotherapy, showing that immune combination chemotherapy does benefit patients with advanced BTC more than single-agent immunotherapy [21, 22]. In a phase II clinical trial (NCT03875235), the difference in efficacy between one ICIs (GP plus durvalumab) in combination with first-line GP regimen chemotherapy and two ICIs (GP plus durvalumab plus tremelimumab) in patients with advanced BTC was investigated. As a result, they found that the ORR of dual immunotherapy combination was even 2% lower than that of single-agent immunotherapy combination; therefore, they concluded that chemotherapy combined with dual immunotherapy did not show significant advantages over single-agent immunotherapy in the first-line treatment of patients with advanced BTC, and even increased the financial cost for patients compared to single-agent immunotherapy. The study also found that the ORR of patients treated with second-line therapy in combination with immunotherapy was 20% lower than that of patients treated with first-line therapy, suggesting that patients benefit more from the combination of ICIs at first-line therapy in the treatment of patients with advanced BTC, This result is consistent with both our retrospective study and the results of the phase III clinical trial of TOPAZ-1 [7, 19, 23].

In a multifactorial regression analysis, we also found that inflammatory indicators such as NLR, PLR, MLR, SII, and CONUT score were independent influences on the prognosis of patients with advanced BTC (all *p* < 0.05), where lower inflammatory indicators and CONUT score were associated with better OS and PFS of patients. Ellegård et al. [24] have also found that the systemic inflammatory response is the most important biochemical indicator in malignant diseases. A large prospective cohort study of the association between systemic inflammatory markers and cancer risk was conducted by Therese Haugdahl Nøst et al. [25] who found that a variety of cancers were positively associated with the risk of SII, NLR, PLR, and MLR by analyzing the association between systemic inflammatory markers and cancer risk in 440,000 patients. Hyerim Ha1 et al. [26] found that patients with high levels of NLR, PLR, and SII had poorer OS by analyzing the hematological indicators of 158 patients with advanced BTC. Chan Su Park et al. [27] also found in a retrospective study that lower MLR was significantly associated with better OS in patients with advanced BTC treated with pembrolizumab in the second line. This result is consistent with the results in the retrospective study we conducted, in which we also found a significant correlation between lower NLR (*p* = 0.0083), PLR (*p* = 0.020), SII (*p* = 0.0161), and MLR (*p* < 0.001) and better OS of patients. Chiao-En Wu et al. [28] also found that patients with a response of CR or PR had significantly lower MLR values than those with a response of SD or PD and concluded that high MLR was an independent influencing factor for poor patient prognosis.

Sorayya Kheirouri et al [29] and Lejia Sun et al [15] both found in their studies that CONUT scores can be used to predict OS, cancer-specific survival (CSS), and (recurrence-free survival (RFS) in cancer patients, which are independent prognostic factors for OS and CSS in patients with multiple cancers, and that higher CONUT scores are associated with lower cancer survival, and the nomogram based on the CONUT score predicts OS in BTC patients and performs better than the American Joint Committee on Cancer (AJCC) staging system. The CONUT score includes total cholesterol concentration, serum albumin concentration, and peripheral blood lymphocyte count. Total serum cholesterol can reflect the body’s lipid metabolism ability, serum albumin can reflect the body’s protein synthesis ability, and peripheral blood lymphocyte count is similar to NLR, PLR, and other inflammation indicators, which can reflect the body’s immune function [30, 31]. However, many believe that the hyposerum albuminemia seen in patients with advanced cancer may be more related to the systemic inflammatory response, and therefore, it is more recommended to consider serum albumin as an indicator of inflammation rather than nutrition in the CONUT score [15, 32]. In addition, a higher CONUT score indicates not only a poor nutritional status but also a degree of impaired immune function, which may be associated with a poorer prognosis, as it has been found that the nutritional status of patients is closely related to the normal functioning of the immune system, and that poor nutritional status leads to immune system dysfunction, with varying degrees of dysfunction of immune cells such as lymphocytes, macrophages and neutrophils [33, 34]. The results of this retrospective study we conducted also showed a significant correlation between better OS and lower CONUT scores in patients with advanced BTC treated with the combination of ICIs (*p* = 0.0051).

Although there have been many studies confirming the prognostic role of inflammatory biomarkers in patients with BTC, there is a paucity of studies on the impact and predictive role of inflammatory indicators and the CONUT score in patients with advanced BTC who receive ICIs in combination with first-line therapy [14, 35]. As far as we know, this is the first study to assess the prognostic relevance of NLR, PLR, MLR, SII, and CONUT scores to patients with advanced BTC receiving first-line ICIs in combination with chemotherapy. We identified NLR, PLR, MLR, SII, and CONUT scores as independent influences on PFS and OS in patients with advanced BTC based on multifactorial regression analysis, which was considered and included in the final nomogram and validated. Both the training and validation cohorts were statistically significant (both *p* < 0.05), with a C-index of 0.77 (95% CI: 0.71–0.84) for the training cohort and 0.75 (95% CI: 0.68–0.81) for the validation cohort for the nomogram. In addition, the AUC values were greater than 0.65 at 12, 18 and 24 months for both the training and validation cohorts (all *p* < 0.05). Our nomograms were also well calibrated and ultimately found to have better clinical prognostic value than any single prognostic factor.

This retrospective study of ours was carried out based on clinical reality and analyzed the effectiveness of combined ICIs in patients with advanced BTC at the time of first-line treatment and produced positive results. The currently known clinical evidence on the use of ICIs for the first-line treatment of patients with advanced BTC is insufficient, as most phase II/III clinical trials are still ongoing, except for the phase III clinical trial of TOPAZ-1, which is already conclusive, so this study we conducted provides a meaningful reference on the feasibility of first-line application of ICIs for patients with advanced BTC. In addition, we developed a nomogram for predicting 12-, 18- and 24-month survival in patients with advanced BTC based on multifactorial analysis; according to us, this nomogram is the first model to predict the prognosis of patients with advanced BTC receiving first-line immune combination therapy, and the model was prospectively validated in the validation cohort and yielded results that were generally consistent with the training cohort. Our nomogram has been calibrated and tested and proven to be realistic and practical, allowing simple and intuitive prediction of patient OS at 12, 18, and 24 months. However, there are also some shortcomings in our study. Studies failed to analyze the adverse effects and safety between immune combination therapy and patients treated with chemotherapy alone; failed to sequence cancer-related genomes or exons to explore possible associations between immunotherapy effects and the genome of advanced BTC to more precisely guide the practical application of immunotherapy in the clinic and to aid the effective screening of target populations. Our nomogram has good predictive power for the entire cohort, but has insufficient risk stratification power for patients in different subgroups; moreover, the nomogram was generated based on clinical data from a single institution in China, without including data from other countries or regions and excluding a large number of data with poor medical records, which may lead to a certain degree of selection bias.

In conclusion, the combination of ICIs with first-line treatment in patients with advanced BTC can effectively prolong the survival of patients. In addition, nomograms based on independent prognostic factors such as CONUT score, NLR, PLR, MLR, and SII can be used to predict the long-term survival of patients with advanced BTC treated with ICIs in the first line, and their predictive ability is better than any single factor, which can more accurately assess the OS of patients with advanced BTC and provide more personalized guidance for patient treatment selection.

### Supplementary Information

Additional file1 (DOCX 16 kb)

## Data Availability

All data and materials are real and available.
