# Supplementary material for: Comparison of the effectiveness of chemotherapy combined with immunotherapy and chemotherapy alone in advanced biliary tract cancer and construction of the nomogram for survival prediction based on the inflammatory index and controlling nutritional status score
Source: Cancer Immunol Immunother. 2023 Sep 5;72(11):3635–49. doi: 10.1007/s00262-023-03513-4 (PMC10576733; doi:10.1007/s00262-023-03513-4)
Supplement: Supplementary file 1 — Additional file1 (DOCX 16 kb) [file 262_2023_3513_MOESM1_ESM.docx]

**Supplementary Table** Cox Univariate and Multivariate Analysis

| **Variables** | **Univariate analysis** |  |  |  | **Multivariate analysis** | |  |
| --- | --- | --- | --- | --- | --- | --- | --- |
|  | **HR** | **HR 95% CI** | ***P value*** |  | **HR** | **95% CI** | ***P value*** |
| Gender |  |  | **0.048** |  |  |  | 0.518 |
| Male | Reference |  |  |  | Reference |  |  |
| Female | 1.680 | 1.005-2.809 |  |  | 1.366 | 0.212–8.820 |  |
| Age（years） |  |  | 0.903 |  |  |  |  |
| ＜60 | Reference |  |  |  |  |  |  |
| ≥60 | 0.998 | 0.794-1.024 |  |  |  |  |  |
| Smoking/Drinking |  |  | **0.022** |  |  |  | 0.242 |
| Yes | Reference |  |  |  | Reference |  |  |
| No | 1.468 | 0.793-2.917 |  |  | 0.76 | 0.520–1.110 |  |
| Tumor location |  |  | 0.397 |  |  |  |  |
| CCA | Reference |  |  |  |  |  |  |
| GCA | 1.007 | 0.842-1.204 |  |  |  |  |  |
| Differentiation |  |  | **0.019** |  |  |  | 0.622 |
| poor | Reference |  |  |  | Reference |  |  |
| Modest-well | 0.806 | 0.634-1.025 |  |  | 1.176 | 0.808–1.433 |  |
| Serum albumin |  |  | **0.008** |  |  |  | 0.171 |
| <36g/L | Reference |  |  |  | Reference |  |  |
| ≥36g/L | 0.467 | 0.266-0.832 |  |  | 0.794 | 0.467–1.113 |  |
| Liver metastasis |  |  | **0.039** |  |  |  | 0.052 |
| Yes | Reference |  |  |  | Reference |  |  |
| No | 1.751 | 1.029-3.001 |  |  | 2.066 | 1.604–3.316 |  |
| Metastasis quantity |  |  | **0.006** |  |  |  | 0.055 |
| Yes | Reference |  |  |  | Reference |  |  |
| No | 2.108 | 1.234-3.603 |  |  | 1.545 | 1.192–2.125 |  |
| Types of ICIs |  |  | 0.328 |  |  |  |  |
| Camrelizumab | Reference |  |  |  |  |  |  |
| Others | 0.915 | 0.766-1.093 |  |  |  |  |  |
| Response |  |  | **0.046** |  |  |  | 0.218 |
| CR-PR | Reference |  |  |  | Reference |  |  |
| SD-PD | 0.661 | 0.433-1.010 |  |  | 0.743 | 0.213–4.816 |  |
| NLR |  |  | **0.010** |  |  |  | **0.021** |
| ＜3.0 | Reference |  |  |  | Reference |  |  |
| ≥3.0 | 1.949 | 1.176-3.230 |  |  | 1.344 | 1.009–2.182 |  |
| PLR |  |  | **0.003** |  |  |  | **0.042** |
| ＜160 | Reference |  |  |  | Reference |  |  |
| ≥160 | 2.249 | 1.327-3.813 |  |  | 1.135 | 0.821–2.058 |  |
| Total bilirubin |  |  | 0.450 |  |  |  |  |
| <21umol/L | Reference |  |  |  |  |  |  |
| ≥21umol/L | 0.795 | 0.438-1.442 |  |  |  |  |  |
| ALT |  |  | 0.311 |  |  |  |  |
| <50U/mL | Reference |  |  |  |  |  |  |
| ≥50U/mL | 1.291 | 0.788-2.115 |  |  |  |  |  |
| CA19-9 |  |  | **0.006** |  |  |  | 0.066 |
| <37U/mL | Reference |  |  |  | Reference |  |  |
| ≥37U/mL | 2.366 | 1.287-4.348 |  |  | 1.005 | 0.701–1.958 |  |
| AST |  |  | 0.100 |  |  |  |  |
| <40U/mL | Reference |  |  |  |  |  |  |
| ≥40U/mL | 1.526 | 0.992-2.526 |  |  |  |  |  |
| MLR |  |  | **<0.001** |  |  |  | **0.001** |
| ＜2.3 | Reference |  |  |  | Reference |  |  |
| ≥2.3 | 3.835 | 2.234-6.583 |  |  | 0.723 | 0.361–1.123 |  |
| SII |  |  | **0.002** |  |  |  | **0.036** |
| ＜830 | Reference |  |  |  | Reference |  |  |
| ≥830 | 2.280 | 1.348-3.856 |  |  | 1.273 | 0.774–1.701 |  |
| CONUT score |  |  | **0.002** |  |  |  | **0.018** |
| ＜2 | Reference |  |  |  | Reference |  |  |
| ≥2 | 2.130 | 1.374-3.884 |  |  | 1.073 | 0.687–1.974 |  |

Notes：HR: hazard ratio; CCA: cholangiocarcinoma; GCA: gallbladder carcinoma; CI: confidence interval; NLR: neutrophil to lymphocyte ratio; PLR: platelet to lymphocyte ratio; MLR: monocyte to lymphocyte ratio; SII: systemic-inflammatory index; ALT: alanine transaminase; AST: aspartate transaminase; CA19-9: glycoprotein antigen 19-9; CONUT score: controlling nutritional status score.
